# Oleanolic acid ameliorates intestinal alterations associated with EAE

**DOI:** 10.1186/s12974-020-02042-6

**Published:** 2020-11-27

**Authors:** Beatriz Gutierrez, Isabel Gallardo, Lorena Ruiz, Yolanda Alvarez, Victoria Cachofeiro, Abelardo Margolles, Marita Hernandez, Maria Luisa Nieto

**Affiliations:** 1 Instituto de Biología y Genética Molecular (IBGM-CSIC/UVa), Valladolid, Spain; 2 Dairy Research Institute of Asturias, Spanish National Research Council (IPLA-CSIC), Paseo Río Linares s/n, Villaviciosa, Asturias Spain; 3 MicroHealth Group, Instituto de Investigación Sanitaria del Principado de Asturias (ISPA), Oviedo, Asturias Spain; 4 Departamento de Fisiología, Facultad de Medicina, Universidad Complutense de Madrid and Instituto de Investigación Sanitaria Gregorio Marañón (IiSGM), Madrid, Spain

**Keywords:** Cytokines, EAE, Intestinal dysfunction, Inflammation, Immune markers, Mucins, Multiple sclerosis, Oleanolic acid, Oxidative stress, Triterpenes

## Abstract

**Background:**

Multiple sclerosis (MS) is a chronic demyelinating autoimmune disease affecting the CNS. Recent studies have indicated that intestinal alterations play key pathogenic roles in the development of autoimmune diseases, including MS. The triterpene oleanolic acid (OA), due to its anti-inflammatory properties, has shown to beneficially influence the severity of the experimental autoimmune encephalomyelitis (EAE), a preclinical model of MS. We herein investigate EAE-associated gut intestinal dysfunction and the effect of OA treatment.

**Methods:**

Mice with MOG_35–55_-induced EAE were treated with OA or vehicle from immunization day and were daily analyzed for clinical deficit. We performed molecular and histological analysis in serum and intestinal tissues to measure oxidative and inflammatory responses. We used Caco-2 and HT29-MTX-E12 cells to elucidate OA in vitro effects.

**Results:**

We found that OA protected from EAE-induced changes in intestinal permeability and preserved the mucin-containing goblet cells along the intestinal tract.

Serum levels of the markers for intestinal barrier damage iFABP and monocyte activation sCD14 were consistently and significantly reduced in OA-treated EAE mice. Beneficial OA effects also included a decrease of pro-inflammatory mediators both in serum and colonic tissue of treated-EAE mice. Moreover, the levels of some immunoregulatory cytokines, the neurotrophic factor GDNF, and the gastrointestinal hormone motilin were preserved in OA-treated EAE mice. Regarding oxidative stress, OA treatment prevented lipid peroxidation and superoxide anion accumulation in intestinal tissue, while inducing the expression of the ROS scavenger Sestrin-3. Furthermore, short-chain fatty acids (SCFA) quantification in the cecal content showed that OA reduced the high iso-valeric acid concentrations detected in EAE-mice. Lastly, using in vitro cell models which mimic the intestinal epithelium, we verified that OA protected against intestinal barrier dysfunction induced by injurious agents produced in both EAE and MS.

**Conclusion:**

These findings reveal that OA ameliorates the gut dysfunction found in EAE mice. OA normalizes the levels of gut mucosal dysfunction markers, as well as the pro- and anti-inflammatory immune bias during EAE, thus reinforcing the idea that OA is a beneficial compound for treating EAE and suggesting that OA may be an interesting candidate to be explored for the treatment of human MS.

**Supplementary Information:**

The online version contains supplementary material available at 10.1186/s12974-020-02042-6.

## Introduction

Multiple sclerosis (MS) is a chronic demyelinating inflammatory disease of the central nervous system (CNS) that mostly affects young adults and is two to three times more common in women than in men. Its common clinical manifestations include visual disturbances, weakness, loss of balance and fatigue, depending on which areas of the CNS are affected [1, 2]. In addition, MS patients frequently display dysregulation of the autonomic nervous system, exhibiting gastrointestinal (GI) dysfunction, including constipation, dysphagia, and fecal incontinence [3].

It is largely accepted that MS develops spontaneously as consequence of an autoimmune response, where effector cells from the immune system infiltrate the patient’s brain and spinal cord leading to destruction of myelin sheaths and subsequent axonal degeneration. Lately, several studies have revealed an increase in intestinal permeability, often referred to as a “leaky gut”, in MS patients and experimental models [4, 5]. The loss of the protective function of epithelial barriers has been hypothesized to result in an increased infiltration and exposure to the luminal microbial components, which may drive immune-inflammatory processes in the host. Recent studies have pointed out that an impaired intestinal barrier function might cause an imbalance between Th1 and Th2 immune responses, which is critical in MS.

Experimental autoimmune encephalomyelitis (EAE) induced with myelin oligodendrocyte glycoprotein (MOG) is an experimental model widely used for the study of MS [6]. The MOG-EAE in rodents closely resembles the clinical and immunopathological features of the human disease, including some intestinal alterations [7].

Given that most immunomodulatory therapies in clinical use possess unwanted side effects, particularly in long-term treatment, finding new treatments with improved safety and tolerability is an ongoing challenge. Significant studies in recent years have focused on the search for naturally occurring immunomodulatory agents. Oleanolic acid (3β-hydroxyolean-12-en-28-oic acid, OA) is one of the major pentacyclic triterpenes that exists in nature either as free acid or as aglycone precursor and is especially prevalent in plants belonging to the Oleaceae family. OA, as well as herbal extracts containing OA, is well known for its diverse bioactivities, including anti-oxidant, anti-tumour, anti-inflammatory, anti-diabetic, anti-microbial, as well as cardioprotective, neuroprotective, hepatoprotective, and hypolipidemic effects [8–12]. Likewise, several experimental approaches have demonstrated that OA is therapeutically effective without apparent side effects [13, 14]. Evidence for the intense interest in OA and OA-related substances is the high number of studies reported, including phases 2 and 3 clinical trials in patients with type 2 diabetes, chronic kidney disease, and pulmonary hypertension, among others [15, 16]. However, no clinical trials in patients with MS have thus far been reported.

In previous studies, we have shown a protective role of OA in the EAE model [10], and we have proposed in the present study to investigate EAE-associated gut injury and to explore in vivo and in vitro the potential beneficial effects of OA administration. Our findings confirmed that OA treatment markedly slowed the unfavorable intestinal alterations caused by EAE induction related to alterations in gut permeability, mucins expression, inflammation, and oxidative stress. We also verified its direct modulatory effects in well characterized in vitro models which mimic the intestinal barrier properties, Caco-2 cell monolayers, and Caco-2/HT29-MTX-E12 co-cultures.

## Material and methods

### Mouse experimental model and treatment

EAE was induced in 8–10-week-old female C57BL/J6 mice (Charles River Laboratories, Barcelona, Spain) with the MOG_(35–55)_ peptide (MEVGWYRSPFSRVVHLYRNGK) as previously described [10]. Briefly, mice were injected subcutaneously at one site of each flank with 200 μg of MOG_(35–55)_peptide (Proteomics Section, Universitat Pompeu Fabra, Barcelona, Spain) in complete Freund’s adjuvant (Sigma Chemical, St. Louis, MO, USA) containing 4 mg/mL *Mycobacterium tuberculosis* H37Ra (Difco Laboratories, Detroit, MI, USA). Additionally, mice received 300 ng of Pertussis toxin (Sigma Chemical, St. Louis, MO, USA) injected intraperitoneally (i.p.) on days 0 and 2. Control mice were immunized in the same manner using PBS in the absence of the peptide. Mice were treated daily either with 50 mg/kg/d OA i.p. or vehicle from immunization day until termination of the experiment: approximately 25–27 days post-immunization, when EAE-mice showed hind limb paralysis and animals were subsequently sacrificed. Food and water access for severely disabled animals was assured. Blood and different parts of the intestinal tract (colon and cecum) were collected. Tissues were frozen at – 80 °C for protein studies or fixed in 4% paraformaldehyde in PBS, followed by paraffin embedding or OCT embedding and frozen.

Body weight and clinical score of EAE were assessed daily in a double-blind manner on a scale of 0 to 4 as detailed in reference [10]: grade 0, no abnormality; grade 0.5, partial loss/reduced tail tone, assessed by inability to curl the distal end of the tail; grade 1, tail atony; grade 1.5, slightly/moderately clumsy gait, impaired righting ability, or combination; grade 2, hind limb weakness; grade 2.5, partial hind limb paralysis; grade 3, complete hind limb paralysis; grade 3.5, complete hind limb paralysis and fore limb weakness; grade 4, tetraplegic.

C57BL/J6 mice were housed in the animal care facility at the Medical School of the University of Valladolid (UVa), Spain, and provided with food and water ad lib. All experimental protocols were reviewed and approved by the Institutional Animal Ethics Committee of the UVa (3008787).

Oleanolic acid (Extrasynthese, Genay Cedex, France) was first dissolved in 10% w/v DMSO and then diluted with PBS for each experiment (the final concentration of DMSO was 2%, v/v)

### Histology: PAS/Alcian Blue Stain

Sections of distal colon from at least five animals of the different experimental groups were fixed and embedded in paraffin to obtain transversal slices of 5 μm thickness. Then, they were stained with Alcian Blue (AB)/periodic acid-Schiff (PAS), using standard histological techniques, for evaluating mucin expression in goblet cells. Acidic mucins stain blue with AB (pH 2.5) and neutral mucins stain pink with PAS, while mixtures of neutral and acidic mucins appear purple.

The sections from all experimental groups were stained in one single batch to ensure that differences in the staining pattern were not due to technical manipulations, thereby making certain the comparability of the different samples. Slides were examined under an optical microscope (Nikon Eclipse 90i, Nikon Instruments, Inc., Amstelveen, The Netherlands). For quantitative analysis, images were acquired from at least three random fields of view per slice and processed using the ImageJ image analysis program (NIH, Bethesda, MD, USA). The area AB/PAS positive was identified as the ratio to the total tissue area.

### Detection of colonic superoxide anion production

Distal colonic segments were collected and the oxidative fluorescent dye dihydroethidium (DHE, Invitrogen Life Technologies, Burlington, Canada) was used to evaluate the intracellular production of superoxide (O_2_^−^) anion as previously described [17]. Briefly, frozen samples cut into 12-μm thickness sections using a cryostat were incubated with 5 μM of DHE for 30 min in a humidified and light-protected chamber at 37 °C. Fluorescence signals were viewed using a fluorescence microscope (Nikon TE2000, Japan) under a × 10 objective (× 100 final magnification) and a × 20 objective (× 200 final magnification).

At least five images of each colon sample were captured for analysis using fixed exposure time for all groups. The intensity of fluorescence signals was quantified using ImageJ software (NIH, Bethesda, MD, USA). A single researcher that was unaware of the experimental groups performed the analysis.

### Biomarker analyses on EAE mouse samples using an enzyme-linked immunosorbent assay (ELISA)

Selected biomarkers were measured in serum and colon tissue samples by ELISA according to the manufacturer’s protocols. Serum was collected at the end of evolution time and immediately stored at – 80 °C. Colon tissues were flushed with PBS, weighed and homogenized (1:10, w/v) in ice-cold PBS supplemented with 0.4 M NaCl, 0.05% Tween 20, 1% EDTA and a protease inhibitor cocktail containing PMSF, leupeptin and aprotinin (Sigma-Aldrich, St Louis, MO, USA), and centrifuged at 10.000 rpm for 10 min at 4 °C. Mouse TNFα, IL-1β, pro-IL-1β, IL-23, and IL-25 ELISA kits from eBioscience (San Diego, CA, USA). Mouse IGF-1 and KC ELISA kits were from Peprotech (Rocky Hill, NJ, USA). Mouse IL-33 and Galectin-3 (Gal-3) DuoSet ELISA Kits were from R&D (R&D Systems, Minneapolis, MN, USA). Mouse Motilin was from Elabscience (Houston, TX, USA). Mouse IL17A and human IL-8 were from Immunotools (Friesoythe, Germany). Mouse GDNF ELISA kit was from Cusabio (Cusabio Biotech CoLtd, Wuhan, China). Mice *n* = 5–7 per group.

### MOG_35–55_-specific IgG quantitation

Levels of antibodies directed against MOG peptide were determined in serum collected from mice on day 25 after immunization using the ELISA technique. 96-well polystyrene microtiter plates were coated with 0.5 μg/well of MOG_35–55_ peptide by overnight incubation in PBS at 4 °C. After blocking with 5% BSA for 1h, the wells were incubated in duplicate with the serum samples diluted 1:60 in PBS for 2 h at room temperature. After washing, a HRP-conjugated goat anti-mouse IgG1 (1:2000) from Serotec (Sigma-Aldrich, St. Louis, MO) was added for 90 min. After another washing, adding the substrate, and arresting the reaction with 0.1 N HCl, absorbance was read at 450 nm. The results were expressed as mean optical density at 450 nm.

### Myeloperoxidase

Myeloperoxidase (MPO) activity was assayed on colonic tissue samples homogenized in 50 mM phosphate buffer with 0.5% hexadecyltrimethylammonium bromide (HDTAB) (1:10, w/v). MPO activity was calculated by ratio of change in absorbance and molar extinction coefficient for TMB (59000 M^−1^ cm^−1^) [18].

### Ex vivo intestinal permeability assay

Ex vivo detection of intestinal permeability was performed following the protocol of Zhong et al. with some modifications [19]. Colon tissue samples were extracted into Krebs-Henseleit bicarbonate solution (KHBB) containing 8.4 mM HEPES, 119 mM NaCl, 4.7 mM KCl, 1.2 mM MgSO_4_, 1.2 mM KH_2_PO_4_, 25 mM NaHCO_3_, 2.5 mM CaCl_2_, and 11 mM glucose (pH 7.4). After that, one end was sutured and 100 μl of fluorescein-labeled dextran-40 (FD-40, MW 40 kDa, 10 mg/ml) was injected using a gavage needle. The other end of the sample was then sutured to form a 5-cm sac. After a quick dip in KHBB to remove the presence of fluorophore on the outside, the intestinal sac was incubated in 2 ml of new buffer, at 37 °C for 20 min. Finally, the fluorescence of the FD-40 transferred from the intestinal lumen to the incubation solution (Ex./Em. 485/530 nm) was measured in a fluorimeter. Intestinal permeability was expressed in micrograms of extravasated FD-40/cm/min.

### Oxidative stress markers

Colon homogenate samples were assessed in duplicates to determine the presence of lipid peroxidation products as malondialdehyde (MDA) concentration, as well as the antioxidant capacity by FRAP assay. These parameters were estimated accordingly to the method of Ohkawa et al. [20] and Benzie et al. [21], respectively. Sestrin-3 levels, a ROS detoxification protein, were measured by a commercial ELISA (eBioscience, San Diego, CA, USA) following the manufacturer’s instructions.

### Western blotting

Protein samples (50 μg) from the homogenized colonic tissue samples were solubilized in Laemmli buffer, boiled for 10 min and separated by SDS-PAGE. Similar loading and transfer of proteins were verified by staining the blots with Ponceau S. Proteins were transferred to polyvinylidene difluoride membranes (Hybond-P; Amersham Biosciences, Piscataway, NJ, USA). Membranes were probed with primary antibody against NLRP6 (sc-50635, Santa Cruz Biotech, Inc.; dilution 1/500) and β-actin (A5441, SIGMA, MO, USA; dilution 1/30000) as a protein loading control. Protein levels were detected with HRP-conjugated secondary antibodies and visualized using the ECL detection system according to the manufacturer’s instructions. Quantification of images was done by scanning densitometry using ImageJ software (NIH, Bethesda, MD, USA). Results are expressed as an n-fold increase over the values of the control group in densitometric arbitrary units.

### In vitro studies

#### Cell culture

The human Caco-2 (kindly provided by Dr. E. Arranz, IBGB-UVa/CSIC, Spain) and HT29-MTX-E12 (kindly provided by Dr. M. Alzheimer, IMIB-Würzburg Universität, Germany) epithelial carcinoma cells (absorptive and goblet cells, respectively) have been used in the present study, since they are an excellent human enterocyte-like model to study intestinal epithelial physiology.

The Caco-2 cells (between passages 19 and 35) were cultured in DMEM (glutamine, high glucose), supplemented with 1% nonessential amino acids (Sigma Chemical Co. St. Louis, MO, USA), 10% FCS, 100 U/ml penicillin and 100 pg/ml streptomycin (Life Technologies, Carlsbad, CA, USA). The cells were incubated in a humidified 5% CO_2_ incubator at 37 °C. The mono-culture of Caco-2 cells formed tight junctions at day 17–21 post-confluence. Differentiated cell layers showing high transepithelial resistance (TEER) values (~ 400–500 Ω × cm^2^), measured with Millicell electrodes (Millicell-ERS, Millipore, Billerica, MA, USA) were used for experiments.

In co-culture experiments, Caco-2 and HT29-MTX-E12 cells were grown separately and were mixed prior to seeding at a ratio 75:25 (to simulate the large intestine) at a final density of 1 × 10^5^ cells/insert onto polycarbonate membrane Transwell inserts with 0.4 μm pore size, 0.33 cm^2^ growth surface in 24-well plates.

#### Cell viability assay

Cell survival was assessed using the Cell Titer 96 Non-Radioactive Cell Proliferation Assay (Promega Corporation, Madison, WI, USA), according to the manufacturer's recommendations. Briefly, serum-starved Caco-2 cells, seeded in 96-well tissue culture plates (10 × 10^3^ cells/well) were treated with either 5–20 μM of OA, 10^−3^–5 mM of iso-valeric acid or vehicle. After 24 h of incubation, formazan product formation was assayed by recording the absorbance at 490 nm in a 96-well plate reader as an assessment of the number of metabolically active cells. Three different assays were each performed in triplicate.

### Immunofluorescent staining, microscopy, and image analysis

Caco-2 cell monolayers grown on 0.33 cm^2^ Transwell supports were fixed with 4% paraformaldehyde in PBS for 30 min, washed, and permeabilized in 0.3% Triton X-100 in PBS. Then, they were incubated with a monoclonal antibody against Zonula occludens-1 (ZO-1; Invitrogen, Carlsbad, CA, USA), washed, and incubated with FITC-conjugated secondary antibody (Invitrogen, Carlsbad, CA, USA) and DAPI. Stained monolayers were examined using a Leica TSC SP5 (Leica Microsystems, Wetzlar, Germany) confocal microscope equipped with × 60 objectives. Stacks were imported into AutoVisualize 9 (AutoQuant Imaging) for presentation of 3D projections and maximum volume projections were created with the 5D viewer deconvolution software.

### Determination of transepithelial electrical resistance (TEER) and permeability of the cell monolayer

#### Transepithelial electrical resistance (TEER) measurement

The integrity of the Caco-2 monolayer and the Caco-2:HT29-MTX co-culture was determined by measuring the TEER value [22]. Cells were grown in 24-well plates and seeded at 1 × 10^5^ cells/insert onto polycarbonate membrane Transwell inserts with 0.4 μm pore size, 0.33 cm^2^ growth surface (Corning, Inc.; Lowell, MA, USA). Cells were cultured for 21 days to reach differentiation. Differentiated and polarized cell layers showing high transepithelial resistance (TEER) values, measured with Millicell electrodes (Millicell-ERS, Millipore, Billerica, MA, USA) were used for experiments. TEER recorded in unseeded Transwell inserts was subtracted from all values.

Confluent and differentiated Caco-2 cell monolayers or Caco-2/HT29-MTX-E12 co-cultures were (i) incubated in the presence of the microbial metabolite iso-valeric acid (apical) for 24 h; or (ii) pretreated with the indicated doses of OA for 30 min (apical) and then stimulated with 100 ng/ml of TNFα for different times (apical or basolateral application, as indicated). To facilitate comparisons between conditions, TEER measures at 24 h were normalized to untreated-control cell and expressed as percentage of control.

#### Permeability studies

Permeability of the cell monolayer was determined by using the macromolecular tracer FITC-labeled Dextran (FD-40, Sigma Chemical Co. St. Louis, MO, USA). Confluent and differentiated Caco-2 cell mono-cultures or Caco-2/HT29-MTX-E12 co-cultures were pretreated with the indicated doses of OA for 30 min (apical) and then stimulated with 100 ng/ml of TNFα for 24 h (apical or basolateral application, as indicated). Then, media was aspirated and both chambers were washed with HBSS. After that, 200 μl of 10 mg/ml FITC-dextran dissolved in HBSS was added at the apical compartment of each insert. After 1 h incubation at 37 °C, 200 μl aliquots were taken from the basolateral chamber and plated into a black, flat bottom 96 well plate. The fluorescence intensity was measured in a Fluoroskan Ascent FL microplate reader (Thermo Electron. Corporation, Waltham, MA, USA) with the setting of 485 nm (excitation) and 530 nm (emission). The amount of FITC-Dextran transported into the basolateral compartment (permeability flux) was extrapolated from a standard curve and expressed as mg/ml.h. Results were expressed as apparent permeability coefficient (Papp) and defined as cm/h. “Papp” is derived from the ratio of flux rate (mg/ml.h) to that of initial concentration (in mg/ml) and surface area of the membrane.

### Analysis of IL-8 production by Caco-2 cell monolayer

Caco-2 cells (1 × 10^5^ cells/well) were seeded into 24-well tissue culture plates and cultured for 48 h. After complete confluence, the cells were (i) incubated in the presence of the indicated doses of iso-valeric acid (apical); or (ii) preincubated with the indicated doses of OA for 30 min at 37 °C, and then stimulated with 25 ng/ml of IL-1β for 24 h at 37 °C. The supernatants were harvested to quantify IL-8 production using a human IL-8 ELISA Ready-Set-Go kit (eBioscience, San Diego, CA, USA). Three different assays were each performed in triplicate.

### Determination of intracellular reactive oxygen species (ROS) levels

For detection of intracellular ROS production, Caco-2 cells seeded in 96-well microplates at 1 × 10^4^/well and after serum starvation, cells were incubated overnight at 37 °C with the indicated doses of iso-valeric acid or OA. Cells were subsequently loaded with 10 μM of DCFH-DA for 30 min at 37 °C. After that, OA-treated cells were stimulated with 500 μM of H_2_O_2_ or 400 μM of tert-butyl hydroperoxide (t-BOOH) in DMEM medium for 60 min. Fluorescent signal was measured at Ex. 485 nm-Em. 530 nm, using a plate reader Fluoroskan Ascent FL (Thermo Electron Corporation, Waltham, MA, USA). Results are expressed as an n-fold increase over the values of the control group.

### Measurement of superoxide anion (O_2_^−^) production

The oxidative fluorescent dye DHE (Invitrogen, Carlsbad, CA, USA) was used to evaluate the production of ion superoxide (O_2_^−^). Caco-2 cell monolayers were incubated for 24 h with either vehicle or 100 ng/ml of TNFα (Immunotools, Friesoythe, Germany) in the presence or absence of 10 μM of OA. Cells were then incubated with 5 μM DHE for 30 min in a light-protected humidified chamber at 37 °C. Cells were analyzed under a × 40 objective with a Nikon Eclipse 90i (Nikon Instruments, Inc. Melville, NY, USA). Three different assays were each performed in duplicated. Representative images are shown.

### Short chain fatty acids analysis (SCFA)

The cecal concentration of the SCFAs acetate, propionate, iso-butyrate, butyrate, iso-valerate, and valerate was quantified by gas chromatography in a system comprised of a 6890NGC module (Agilent Technologies Inc., Palo Alto, CA, USA) connected to a flame ionization detector and a mass spectrometry 5973 N detector (Agilent), as previously described [23]. Briefly, samples were obtained from cecal content homogenates, prepared as a 1:5 dilution in PBS (w/v), and 100 μl of cell free-supernatants from the homogenates were mixed with 450 μl methanol, 50 μl internal standard solution (2-ethylbutyric 1.05 mg/ml), and 50 μl 20% v/v formic acid. Supernatants obtained following centrifugation of this mixture were used for SCFA quantification by GC.

### Statistical analysis

All quantified data are expressed as mean ± SEM. GraphPad Prism Version 5.00 software (San Diego, CA, USA) was used to calculate significance. Data were analyzed using one-way ANOVA. Bonferroni test was used for post hoc comparisons among groups. The Pearson correlation coefficient was applied to assess the relationship between selected biomarkers and EAE severity. *p* ≤ 0.05 was considered statistically significant.

## Results

### Effects of OA on clinical signs, colon length, and histopathology in EAE mice

To study the impact of OA treatment in the pathogenesis of EAE-induced intestinal injury, we evaluated the severity of intestinal damage in EAE mice treated with OA according to the protocol shown in Figure S1A. When immunized by MOG_35–55_ peptide, C57BL/J6 mice developed the classical disease characterized by a progressive ascendant paralysis (Fig. S1B). In agreement with previous studies, when OA was daily administered from the day of induction, mice developed less severe signs of motor impairment; in fact, mice developed only minimal pathological abnormalities compared with untreated EAE-animals. Accordingly, the levels of anti-MOG_35–55_ IgG1, a marker of humoral immune reaction, in OA-treated EAE mice were significantly lower than in untreated EAE mice (Figure S1C).

**Fig. 1:**
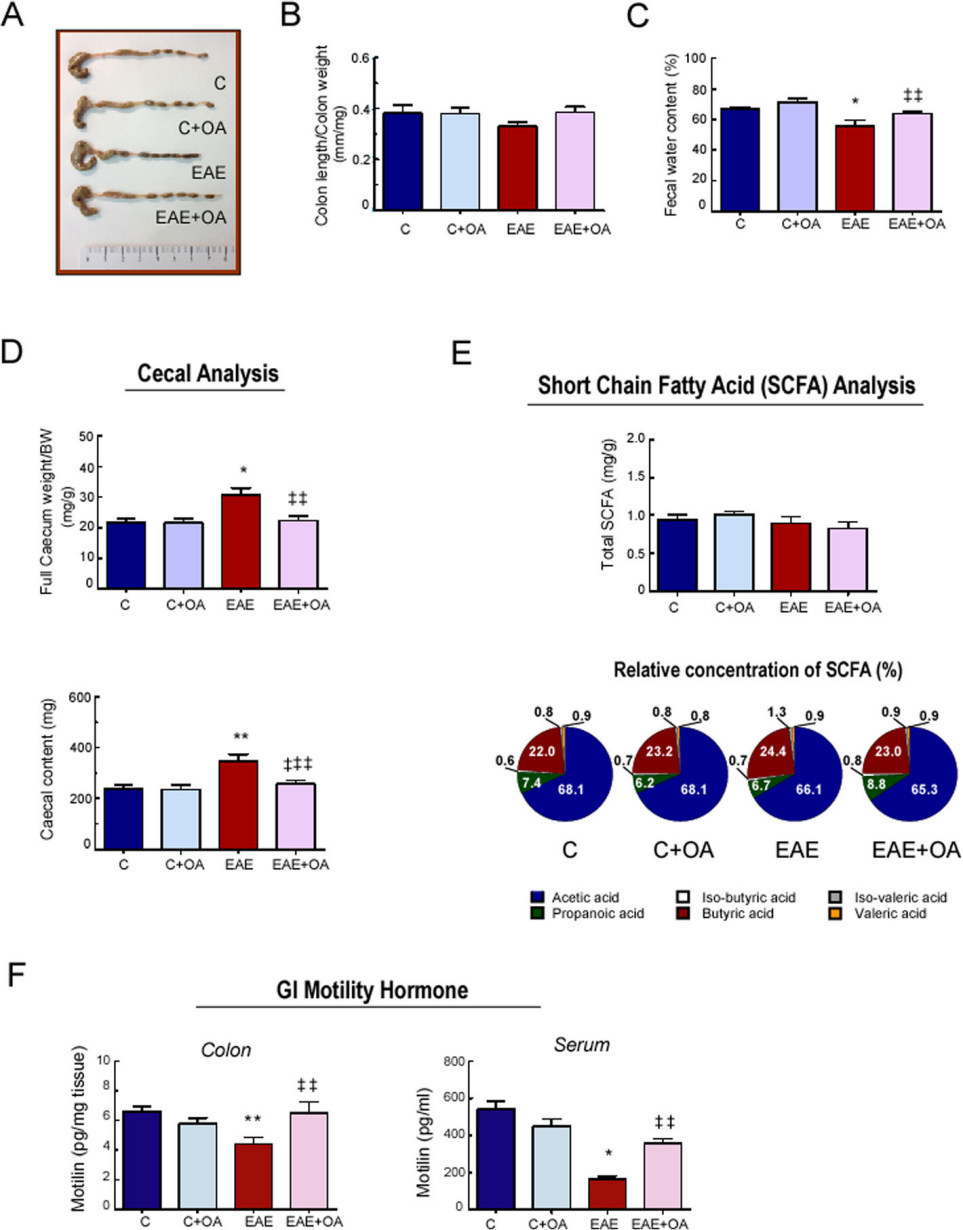
Effect of OA treatment on colon length, fecal parameters and Histopathology in EAE mice. **a** Representative image of colons. **b** Colon length/weight ratio (colon were excised from anus to cecum, measured, and emptied before being weighed). **c** Percentage of water in fecal pellets collected from mice over one hour period. **d** Full cecum weight/body weight ratio and weight of cecal content. **e** Total and relative short-chain fatty acid (SCFA) concentrations in cecal contents. **f** Motillin protein concentration measured in colon extracts and serum samples, from mice of the indicated groups at day 23 post-immunization. Results were expressed as the mean ± SEM, *n* = 5–7 per group. *p < 0.001 and **p < 0.01 vs control; and ^‡‡^*p* < 0.01 and ^‡‡‡^*p* < 0.05 vs untreated-EAE. C, healthy mice. C + OA, healthy mice treated with OA. EAE, induced mice. EAE + OA, induced-mice treated with OA

Macroscopic intestinal analysis did not reveal significant differences, neither in the colon length, nor in the ratio colon length/body weight among mice of the different experimental groups (Fig. 1b, c). Fecal examination showed a significantly lower water content in samples from the EAE group (13% decrease, *p* < 0.001) compared to the control group, while water content was higher in feces of the OA-treated EAE mice: the fecal water content only decreased around a 3% compared to the untreated EAE group (Fig. 1d). Moreover, the cecal luminal content and the full cecum weight/body weight ratio were higher in EAE mice, while OA treatment prevented this increase (Fig. 1e).

Next, total SCFAs were assessed in the cecal content (Fig. 1f). Concentrations of total fecal SCFAs (normalized to cecal weight) did not differ significantly among the four experimental groups. The composition of the individual SCFAs (acetic, propionic, butyric, valeric, iso-butyric and iso-valeric acids) was expressed as a percentage of the total cecal SCFA concentration. A significant increase in propionate abundance was observed in OA-treated EAE mice (*p* < 0.05) (Figure S2A), and the proportion of iso-valeric acid was found significantly increased in the EAE group when compared to controls (*p* < 0.001) (Fig. 1f and Figure S2A and B). Since iso-valeric acid has been related to colon smooth muscle cell relaxation [24], we considered the study of this microbial metabolite in intestinal epithelium, through the different in vitro responses. Further in this line, motilin, an important protein associated with gastrointestinal motility, was also quantified (Fig. 1g). Serum motilin levels were significantly lower in the EAE group than in control group (*p* < 0.001) and significantly higher in the OA-treated EAE group compared to the untreated-EAE mice (*p* < 0.01). Levels of motilin were also significantly lower in the colon of EAE mice than in those of controls, whereas OA treatment prevented this decrease. The concentration of motilin in OA-treated control mice did not differ from that found in untreated controls, neither in serum nor in colon.

### Effect of OA on EAE-induced intestinal oxidative stress

**Fig. 2:**
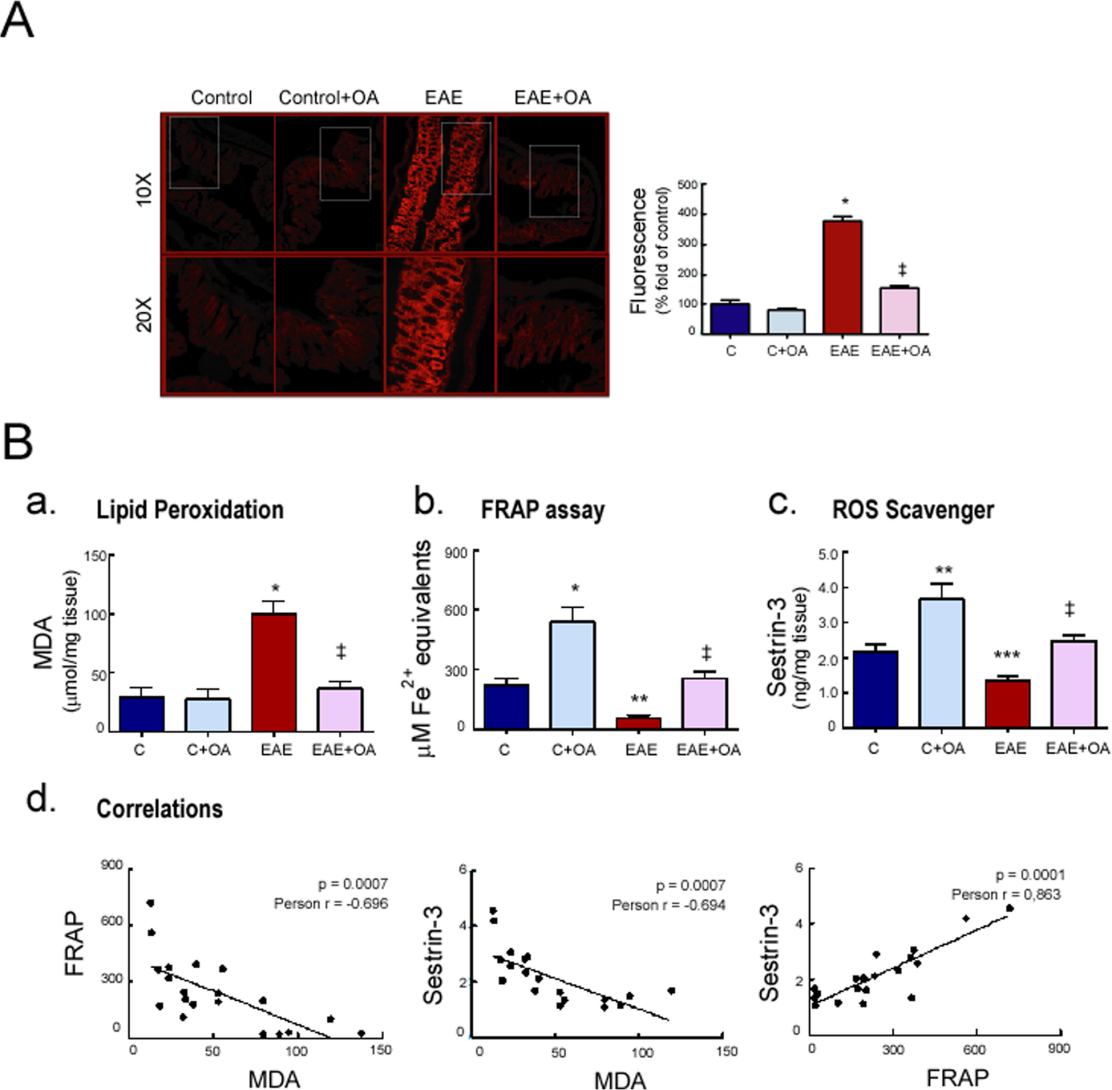
OA treatment reduces oxidative stress in colon tissue from EAE mice. Representative photomicrographs of **a** colon tissue stained with DHE. Histological analysis by fluorescence microscopy and quantification. Objective lens × 10 and × 20. **b** Expression levels in colon of (a) malondialdehyde, MDA, (b) ferric reducing/antioxidant power, FRAP, and (c) the ROS disruptor Sestrin-3. (c, d) Scatter plots of the correlation between oxidative parameters. Results were expressed as the mean ± SEM, *n* = 5–7 per group. *p < 0.001, **p < 0.01, and ***p < 0.05 vs control; and ^‡^*p* < 0.001 vs untreated-EAE. C, healthy mice. C + OA, healthy mice treated with OA. EAE, induced mice. EAE + OA, induced-mice treated with OA

The generation of ROS was evaluated in colon by measuring superoxide anion (O_2_^−^) accumulation in situ using the DHE stain (Fig. 2a). Elevated red fluorescence in colon sections from EAE mice compared to control mice (*p* < 0.001) indicated excess superoxide levels and, as expected, OA treatment prevented these increased O_2_^−^ accumulations (*p* < 0.001).

To obtain more direct evidence for an augmented intestinal oxidative damage in EAE mice, we also assessed some markers of oxidative stress, including MDA (as a lipid peroxidation biomarker), FRAP (as a marker of non-enzymatic antioxidant status) and Sestrin-3 (as a ROS disruptor) in colon homogenates. Our data showed that MDA levels were significantly increased in colon tissue of EAE mice compared with healthy control mice (*p* < 0.001), and OA treatment markedly diminished the enhanced MDA production of EAE mice (Fig. 2b(a)). In contrast, FRAP and Sestrin-3 levels were notably lower in colon from EAE mice compared to controls (*p* < 0.01), and OA treatment increased their levels compared to untreated EAE mice (*p* < 0.001). Interestingly, higher levels of FRAP and sestrin-3 in healthy mice were also observed in the OA-treated group compared with placebo-treated animals (*p* < 0.001) (Fig. 2b(b and c)). A significant inverse correlation was observed between levels of MDA—FRAP and MDA—Sestrin-3, while FRAP—Sestrin-3 was found to be significantly and positively correlated (Fig. 2b(d)). Other pairwise analysis between the oxidative stress markers (MDA, FRAP, and Sestrin-3) with the clinical severity score, also had significant correlation (data not shown)

### Effect of OA on EAE-induced changes in intestinal permeability and mucus status

**Fig. 3.**
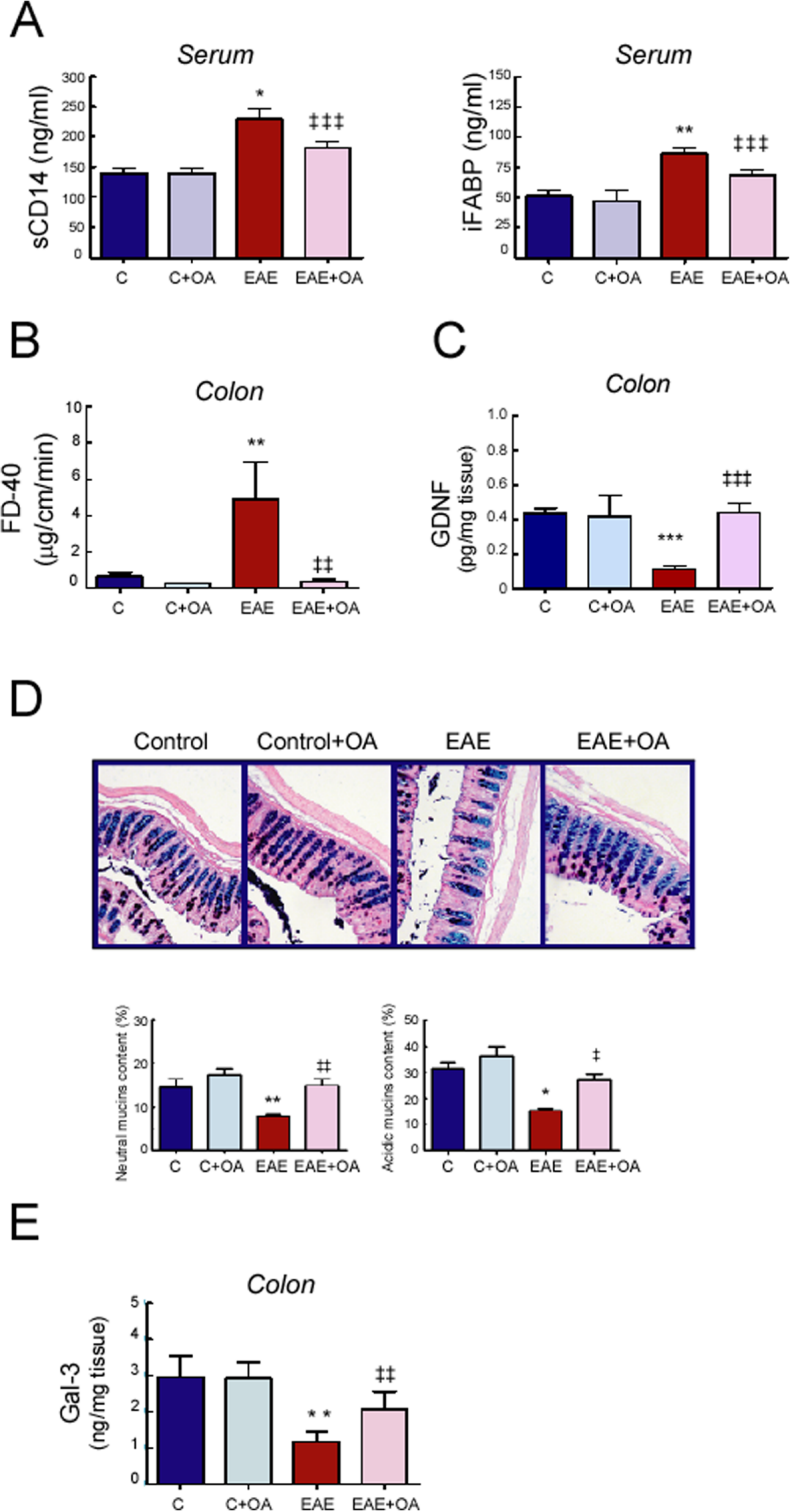
OA treatment protects from intestinal permeability and mucus alteration in EAE mice. **a** Serum levels of soluble CD14 and iFABP were quantified by ELISA. **b** Intestinal sacs prepared from colon to assess intestinal permeability. Sacs were loaded with FITC-labeled Dextran (FD-40) and placed in a bath. After 120 min, the FD-40 concentration from the bath solutions was quantified. **c** Expression of GDNF in colon tissue quantified by ELISA. **d** Histological analysis of colon mucins stained with Alcian Blue/Periodic acid-Schiff (AB/PAS). Objective lens × 10, × 20, and × 20, respectively. Representative photomicrographs (left), quantification graphs (right). **e** Expression of galectin-3 (Gal-3) in colon tissue quantified by ELISA. Results were expressed as the mean ± SEM, *n* = 5–7 per group. *p < 0.001 and **p < 0.01 vs control; and ^‡^*p* < 0.001, ^‡‡^*p* < 0.01, and ^‡‡‡^*p* < 0.05 vs untreated-EAE. C, healthy mice. C + OA, healthy mice treated with OA. EAE, induced mice. EAE + OA, induced-mice treated with OA

Given that intestinal damage due to physiological stressors, such as oxidative stress, may contribute to enhancing intestinal barrier disruption, we assessed in this study changes in the surrogate serological markers of impaired intestinal permeability and microbial translocation, the intestinal fatty acid-binding protein (iFABP) and sCD14 in the serum of untreated and OA-treated EAE mice. Our data showed that EAE induction significantly increased the serum iFABP and sCD14 levels compared with healthy-control mice (*p* < 0.01 and *p* < 0.001, respectively), and OA treatment significantly attenuated this response (Fig. 3a). Significant and direct correlations were found between serum levels of sCD14 and iFABP (*r* = 0.767, *p* = 0.0001), as well as between these parameters and the clinical signs score (*r* = 0.739, *p* < 0.001 and *r* = 0.735, *p* < 0.001, respectively, not shown). Significant correlation of these parameters with the oxidative stress markers analyzed in our study was also detected. Significant negative correlations were found between iFABP—FRAP (*r* = − 0.526, *p* < 0.017) and sCD14—FRAP (*r* = − 0.568, *p* < 0.016), while iFABP—MDA (*r* = 0.784, *p* < 0.0001) sCD14—MDA (*r* = 0.689, *p* < 0.0016) were found to be significantly and positively correlated (plots not shown).

We next determined the effect of EAE induction, as well as the impact of OA treatment, on intestinal barrier function by ex vivo measuring of the intestinal permeability to FD-40 using a non-everted gut sac assay. As shown in Fig. 3b, colonic permeability to FD-40 in the EAE group was significantly higher when compared to a control group (*p* < 0.01), and treatment with OA protected against this increase. Accordingly, the main mediator secreted by enteric glial cells, the glial-derived neurotrophic factor (GDNF), which plays a protective role in the intestinal barrier function, was found to be downregulated in colon of EAE mice, and OA treatment restored its levels to those of healthy-control mice (Fig. 3c).

Finally, given that high levels of oxidative stress in colon have been directly related to a reduction in the content of the different types of mucins in certain pathological situations [25], histological analysis was used to determine this fact in EAE mice. We used the combined analysis of AB/PAS staining to evaluate the variation in acidic and neutral mucin content in colon of mice of the four experimental groups. As shown in Fig. 3d, we observed predominance of acid mucins over neutral mucins in the evaluated slides prepared with colon tissue. Colon sections from EAE mice showed a substantial drop-off in the overall AB/PAS staining when compared with the control group, and intervention with the triterpene OA prevented this significant decrease observed in the tissues from EAE mice. The treatment with OA did not affect the amount of acidic-positive and neutral-positive goblet cells stained in the colon of mice from the control group. Although there was a significant reduction in the expression of both types of mucin in the studied sections of intestinal tracts of EAE mice, the proportion between acidic and neutral mucin species between the healthy control and EAE mice kept constant at ~ 2:1. In keeping with the protective barrier function of mucins, we found a significant direct correlation between colonic goblet cells containing mucins and FRAP (*r* = 0.636, *p* < 0.035)

We also evaluated the expression levels of the endogenous galactoside-binding protein, galectin-3 (Gal-3), which is known to interact with colon mucins and plays an important role in maintaining mucosal barrier function. As shown in Fig. 3e, low Gal-3 levels were decreased in colon from EAE mice, compared with healthy control mice (*p* < 0.01), whereas treatment with OA prevented from this reduction.

### Effect of OA on inflammatory changes in EAE mice intestine

**Fig. 4:**
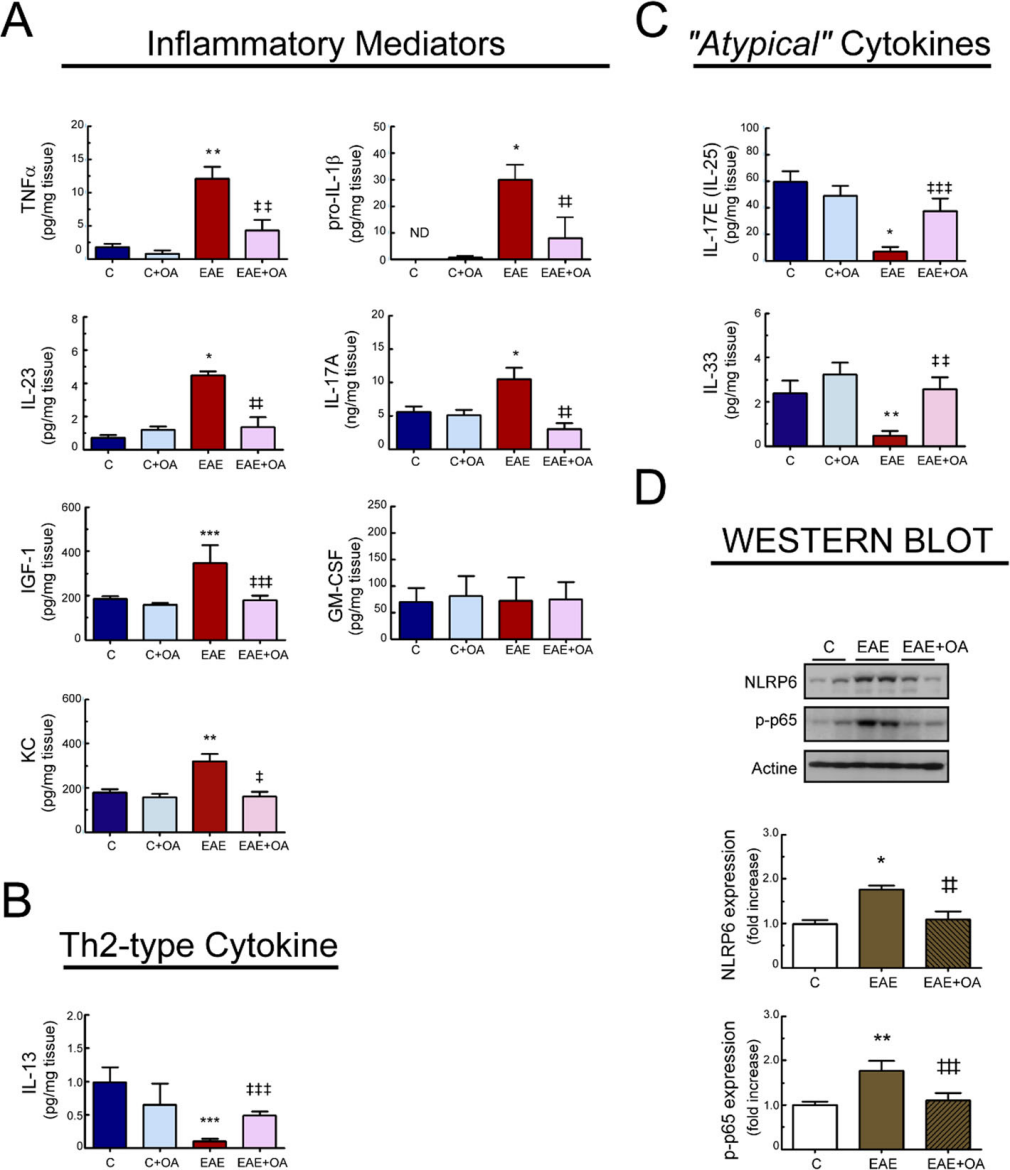
OA treatment modulates inflammatory parameters in colon tissue from EAE mice. Levels in colon of **a** the inflammatory mediators TNFα, IL-1β, IL-23, IL-17, IGF-1, GM-SCF, and KC. **b** The Th2-type cytokine IL-23 and **c** the atypical cytokines IL-25 and IL-33 quantified by ELISAs. **d** Protein expression of NLRP6 and p-P65-NFκB in colon assessed by western blot. Bar graphs represent the mean ± SEM of 5–7 animals. *p < 0.001, **p < 0.01, and ***p < 0.05 vs control; and ^‡^*p* < 0.001, ^‡‡^*p* < 0.01, and ^‡‡‡^*p* < 0.05 vs untreated-EAE. C, healthy mice. C + OA, healthy mice treated with OA. EAE, induced mice. EAE + OA, induced-mice treated with OA

After showing that OA protects from intestinal permeability breakdown in EAE mice, we wondered whether triterpene treatment would also prevent the altered expression of inflammatory mediators that contributes to the pathogenesis of EAE, triggering a cytokine bias mainly associated with protection or recovery from disease. Colon tissue and serum from mice treated with either vehicle or OA were therefore assessed for inflammatory markers relevant to the differentiation of or produced by specific Th cells subsets. We found that OA significantly reduced the levels of the cytokines TNFα, IL-1β, IL-23 and IL-17A, the chemokine KC, as well as the growth factor IGF-1, all of them known to be pro-inflammatory, and observed up-regulated in colon tissue from EAE mice (Fig. 4a). Colon samples from the different experimental groups were also positive for the hematopoietic growth factor and immune modulator, granulocyte-macrophage colony stimulating factor (GM-SCF), but its levels did not differ among groups. Additionally, myeloperoxidase (MPO) activity in colon tissues from EAE mice was ~ 10 times higher compared to control mice, and OA treatment reverses this increase (data not shown), thus confirming that the triterpene treatment prevents the increased number of inflammatory cells in the colon. Likewise, TNFα, IL-1β, Gal-3, and GM-CSF were also observed to be up-regulated in EAE mice serum, compared to healthy-control mice; and OA treatment ameliorated this increased production (Figure S3). Surprisingly, levels of IGF-1 were observed down-regulated in serum from EAE mice, and OA treatment protected from this decrease (Figure S3).

Moreover, we observed that the expression of the signaling molecules NLRP6 and phospho-NFKB-p65 were significantly elevated in EAE mice colon matter compared with controls and OA-treated EAE mice (Fig. 4d).

We also quantified the expression levels of the Th2-type cytokine IL-13 as well as of the IL-33 and IL-17E, two potent type-2 inducing cytokines. The expression levels of these cytokines were downregulated in colon from EAE mice, and OA treatment protected against this decrease.

### In vitro effects of OA or iso-valeric acid on the regulation of barrier integrity in human intestinal epithelial cells

#### Oxidative stress response

**Fig. 5.**
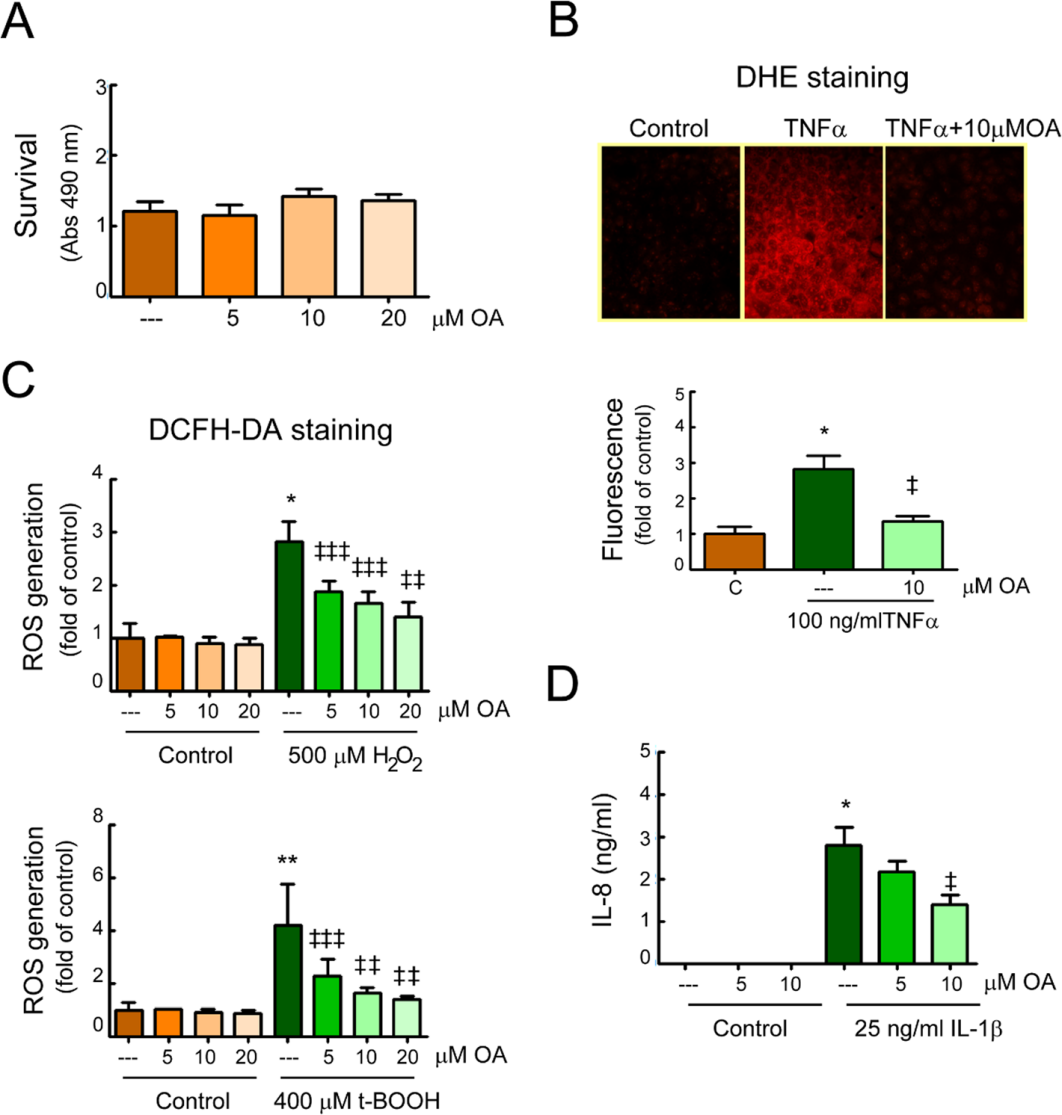
OA treatment inhibits responses of activated intestinal epithelial cells. Caco-2 monolayers were treated for 24 h with the indicated doses of OA and viability was measured (**a**). Caco-2 monolayers, pretreated for 30 min with different doses of OA, were incubated with the indicated stimuli. **b** After 24 h, cells were stained with DHE and superoxide anion accumulation was analyzed by fluorescence microscopy. Representative microphotographs (objective lens × 40) and quantification. **c** After 1 h, cells were stained with DCFH-DA and intracellular ROS-production was analyzed in a microplate reader. **d** After 24 h, IL-8 concentration in the cell-culture supernatant was measured by commercial ELISA. The assays were performed in duplicates, *n* = 3. Results were expressed as the mean ± SEM. *p < 0.001 and **p < 0.01 vs control; and ^‡^*p* < 0.001, ^‡‡^*p* < 0.01, and ^‡‡‡^*p* < 0.05 vs stimuli without OA

We evaluated whether the promising intestinal effects observed in OA-treated EAE mice also involved direct actions on cells that are essential for maintaining a functional intestinal barrier. Therefore, we examined the effects of OA in mono-cultures of Caco-2 cells, a human epithelial cell line that has been widely used as a model of the intestinal epithelial barrier. We treated Caco-2 cell monolayers with the oxidants hydrogen peroxide (H_2_O_2_) and tert-butyl hydroperoxide (t-BOOH), as well as with some relevant inflammatory cytokines such as TNFα and IL-1β, which were found to be enhanced in the EAE mice model. Firstly, we demonstrated that the presence of OA had no significant influence on the viability of Caco-2 cells (Fig. 5a). Then, we evaluated the ability of OA to protect Caco-2 cell monolayers from oxidative stress by using the DHE and DCFH-DA probes. TNFα stimulation generated intracellular O_2_^−^ production in Caco-2 cells, as indicated by the increased red fluorescence, and OA pretreatment abolished this response (Fig. 5b). Likewise, a notable ROS accumulation on H_2_O_2_- and t-BOOH-stimulated Caco-2 cells was observed, compared to untreated ones, which was also diminished by the presence of OA before addition of the agonists (Fig. 5c).

Additionally, to examine the impact of the bacterial by-product, iso-valeric acid in the intestinal epithelium, Caco-2 cells were incubated with iso-valeric acid at different concentrations. Also this time, the viability of cells was not influenced by this branched short-chain fatty acid, and we found no production of reactive oxygen species at any of the doses used (Figure S4A and B)

#### Proinflammatory response

We also investigated the ability of OA to regulate the expression of proinflammatory and chemotactic cytokines, such as IL-8. As shown in Fig. 5d, stimulation of Caco-2 cells with IL-1β led to a strong increase in the production of IL-8, whereas the presence of OA inhibited the up-regulation of this chemokine in a dose-dependent manner.

In addition, the effect of iso-valeric acid on IL-8 release by Caco-2 cells was studied. As shown in Figure S4C, following iso-valeric acid stimulation, IL-8 secretion was significantly increased in Caco-2 cells.

#### Permeability

**Fig. 6:**
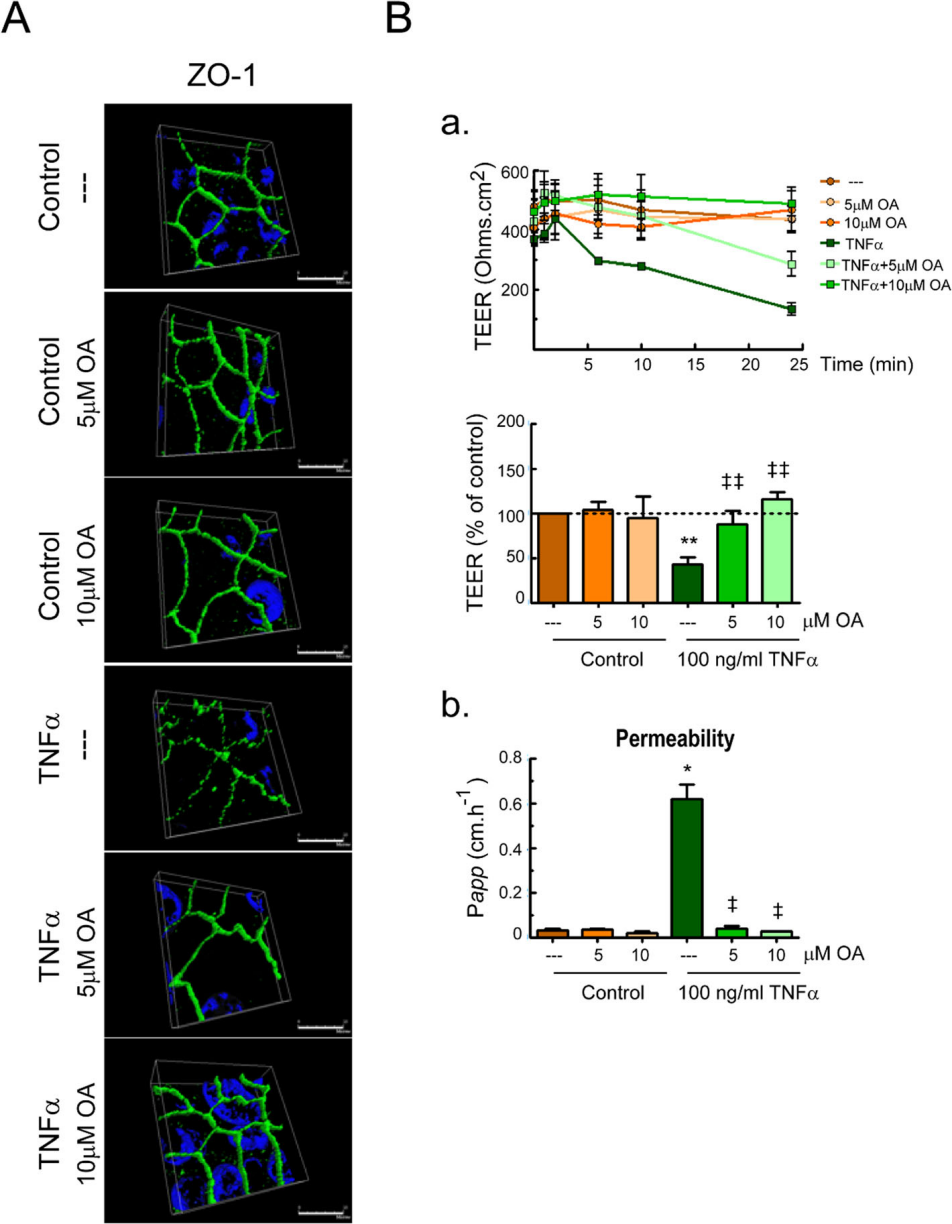
OA treatment modulated intestinal barrier function in differentiated Caco-2 cell monolayers subjected to an inflammatory stimulus. Caco-2 cells were pretreated for 30 min with different doses of OA, and then stimulated with 100 ng/ml of TNFα for 24 h. **a** Representative immunofluorescence images of ZO-1. The three-dimensional projections show the ZO-1 profiles under the indicated treatments. **b** (a) Transepithelial electrical resistance (TEER) was tested at different time points, up to 24 h. TEER values at 24 h normalized to the untreated control (100%) are also showed. **b** (b) Transportation of FITC-dextran (FD-40) from apical to basolateral wells. The assay were performed in triplicates, *n* = 3. Results were expressed as the mean ± SEM. *p < 0.001 and **p < 0.01 vs control; and ^‡^*p* < 0.001 and ^‡‡^*p* < 0.01 vs stimuli without OA

Moreover, we studied the effect of OA on the dysfunction of tight junction barrier integrity induced by TNFα. Previous investigations have shown that TNFα disrupts the localization of tight junction components, such as zonula occludens (ZO)-1 proteins, which leads to a drop-off in the barrier function in Caco-2 cells [26]. As shown in Fig. 6a, we confirmed that TNFα treatment induced a change in ZO-1 distribution in Caco-2 cells, as revealed by immunofluorescence microscopy analysis. Untreated Caco-2 monolayers showed ZO-1 distribution as continuous belt-like structures encircling the cells at the cellular borders, and TNFα treatment disrupted this normal pattern, causing a zig-zagging appearance. However, OA co-treatment prevented the TNFα-induced alteration in the junctional localization of ZO-1 and the characteristic chicken-wire staining pattern was preserved.

Next, the effect of OA on Caco-2 epithelial barrier function was assessed by measurements of TEER and FD-40 permeability (Fig. 6b(a and b)). The presence of OA at 5 and 10 μM did not affect Caco-2 epithelial barrier function. However, TNFα stimulation induced a significant decrease in TEER and a significant increase in FD-40 permeability on Caco-2 cells. As expected, cell pretreatment with OA attenuated the epithelial barrier dysfunction induced by TNFα.

**Fig. 7:**
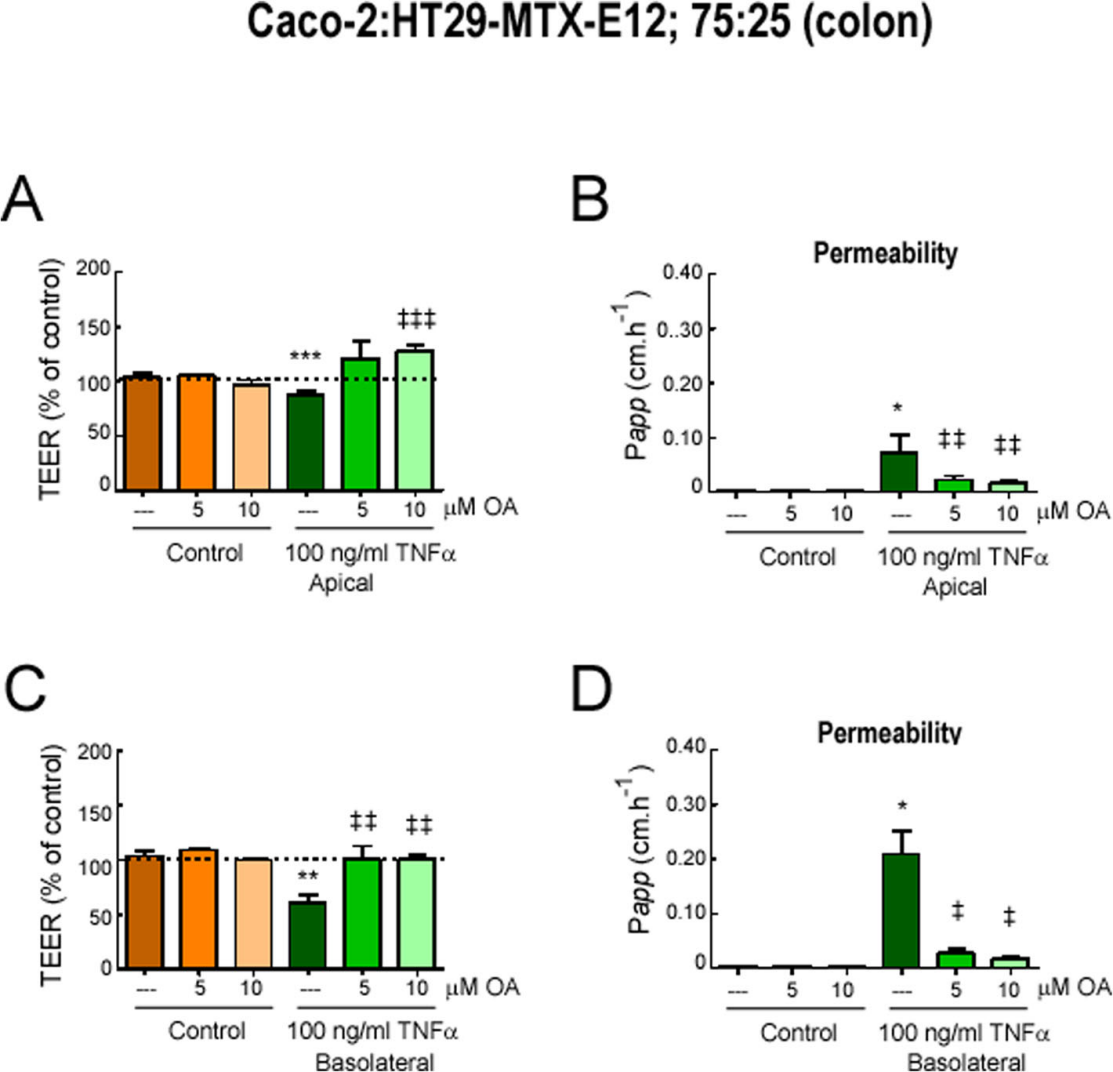
OA treatment modulated intestinal barrier function in differentiated Caco-2:HT29-MTX-E12 co-cultures subjected to an inflammatory stimulus. Differentiated Caco-2:HT29-MTX-E12 co-cultures at 75:25 proportions were stimulated with apical 100 ng/ml of TNFα (**a**, **b**) or basolateral 100 ng/ml of TNFα (**c**, **d**) for 24 h. **a**, **d** TEER values normalized to the untreated control; **b**, **d** FITC-dextran (FD-40) transport. The assays were performed in triplicates, *n* = 3. Results were expressed as the mean ± SEM. *p < 0.001 and **p < 0.01 vs control; and ^‡^*p* < 0.001, ^‡‡^*p* < 0.01, and ^‡‡‡^*p* < 0.05 vs stimuli without OA

Additionally, another in vitro model composed of Caco-2 and HT29-MTX-E12 co-cultures, which represent absorptive and goblet cells, respectively, at the physiologically relevant ratio of 75:25 (large intestine) was used to evaluate the effect of OA on the intestinal cell barrier integrity. The permeability properties of co-cultures were evaluated 21 days after confluence. Figure 7a, c shows TEER measurements across co-cultures. Incubating Caco-2/HT29-MTX-E12 cell monolayers with TNFα (either apical or basolateral) decreased TEER values in comparison with untreated control, and OA pre-treatment protected from this decrease in TEER in a dose-dependent manner. Paracellular permeability to FD-40 was also investigated. As shown in Fig. 7b, d, all TNFα-treated co-cultures displayed an increase in FD-40 permeability, compared to non-treated co-cultures, and OA pre-treatment protected from this paracellular leakage of FD-40 in TNFα-treated cells.

The trans-epithelial electrical resistance (TEER) variance was also analyzed in the presence of iso-valeric acid, finding no alteration induced by this microbial product (Figure S4D).

## Discussion

In this work, we explored EAE-induced disturbances in gut permeability, intestinal inflammation, and oxidative stress, and we investigated the potential beneficial effects of OA treatment. We demonstrated that OA reduced the presence in serum of markers of intestinal barrier disruption such as iFABP and sCD14, preserved the expression levels of the GI hormone motilin, attenuated the depletion of goblet cells containing mucins and improved the impaired intestinal barrier function induced by EAE. Treatment with OA also reduced the indices of inflammation and autoimmunity in colon tissues, (i.e., increased TNFα, IL-1β, IL-23, IL-17A, and KC; and reduced IL-13, IL-33, and IL-25). Moreover, OA inhibited intestinal oxidative stress by reducing superoxide anion accumulation in the colon, and by enhancing the antioxidant capacity and the levels of the ROS disruptor sestrin-3. In previous studies of our group in another model of autoimmune disease, experimental autoimmune myocarditis [12], we observed that OA-treatment significantly increased the percent of Treg compared with those of the control mice group, without affecting the frequency of CD3+, CD4+, and CD8+ T cells, CD14+ macrophages/monocytes, and CD19+ B cells. These results highlight that OA has profound effects in the prevention of this degenerative disease not only by restraining clinical symptoms, such as paralysis of the limbs, production of specific autoantibodies, and BBB dysfunction, as we previously demonstrated [10], but also by protecting against disruption of the intestinal homeostasis.

The usual assessment of symptoms in MS patients has traditionally focused on the skeletal muscle alterations that restrict mobility, while gastrointestinal disorders are often overlooked in MS

Although secondary to spinal cord involvement by the disease, patients with MS frequently refer multiple GI symptoms [3]. Studies of irritable bowel syndrome or functional diarrhea have shown abnormal levels of motilin and water content of feces, which are reverted by therapeutic intervention [27–29]. We found serum motilin and fecal water content markedly decreased in EAE mice compared to control, and OA treatment significantly improved these alterations. Based on these data, one might expect changes in motilin production in MS patients, as found in other GI hormones [30], and possibly a different motilin availability could contribute to the GI symptoms in MS. This hypothesis deserves to be investigated.

Recent evidence suggests that disruption of the intestinal homeostasis may be an early site of MS disease [5, 7]. Intestinal homeostasis is maintained through interactions of the intestinal mucosa, oxidative status, and local and systemic immune-inflammatory factors, among others.

In our study, we observed that OA administration to EAE mice significantly improved some markers of intestinal integrity, such as iFABP and sCD14, found elevated in EAE. CD14 is released primarily from monocytes upon activation, and is considered an indirect marker of bacterial translocation [31]. iFABP, specifically expressed in intestinal epithelial cells, is released into the circulation following enterocyte death, with its plasma concentration being associated with intestinal injury [32]. In line with this, we observed a correlation between the serum iFABP and sCD14 levels, and the severity of the clinical symptoms. iFABP is responsible for the delivery of long-chain fatty acids to mitochondria, which are associated with increased ROS formation [33, 34]. Interestingly, we also observed that the concentrations of iFABP in intestinal tissue from EAE mice tended to be increased, possibly promoting mitochondrial ROS and, in consequence, oxidative process in the gut from EAE mice. The fact that treatment with OA normalized the levels of iFABP, and also reduced gut oxidative stress, further supports this role. The same pattern has also been detected in small bowel tissue from severe intestinal inflammatory diseases [35, 36].

Moreover, the role of OA as a barrier protector was further confirmed, since the permeability of FD40 across intestine was prevented in OA-treated EAE mice. An augmented intestinal permeability could allow luminal antigens to penetrate the intestinal tissue. This, in turn, would activate the immune response and trigger immune-mediated illnesses in different systems, even distant from the gastrointestinal tract, such as CNS.

Emerging evidence indicates that SCFAs produced from gut microbial metabolism play a critical role in regulating the integrity of the epithelial barrier, as well as in neuroimmune homeostasis [37]. Our study showed that, while total SCFAs concentration was similar in the different experimental groups, iso-valeric acid, a volatile fatty acid able to interfere with synaptic neurotransmitter release and previously associated with human depression [38], was more abundant in the EAE group. Depressive symptoms and depressive-like behavior are frequently perceived in both patients and experimental models of MS [39, 40]. Moreover, iso-valeric acid has also been found to block motility of colon smooth muscle cells [24], therefore directly contributing to constipation, one of the clinical signs in EAE and MS. Since we found IL-8 upon stimulation of colon epithelial cells with iso-valeric acid, this SCFA could contribute to the inflammatory environment of EAE, deserving further investigation. Among the three predominant SCFAs (acetic acid, butyric acid, and propionic acid), a significant increase in the abundance of the propionic acid was detected only in the EAE-mice that received OA treatment. Indeed, other studies have reported an increase in some SCFA, including propionic acid, in association with an amelioration of EAE-induced symptoms following a high fiber diet, and SCFA administration, including propionic acid, ameliorated the EAE-induced disease manifestations [41]. Cecal SCFAs formation largely depends on food patterns. In our study, the regular diet (without an essential substrate for SCFA formation) could explain the lack of major differences in SCFA production/abundance between groups, despite the possible difference in the fermentation capacity (different microbiota composition) [42]. Actually, alterations in the gut microbial populations of MS patients, including alterations in SCFA-producing bacterial groups, have already been described [43].

Another important component of the intestinal barrier are mucins; synthesized, stored, and secreted mainly by goblet cells, form a protective mucous layer in intestinal lumen [44]. Notorious goblet cell mucin depletion was observed along the intestinal tract in EAE-mice, which in turn could contribute to the intense inflammatory response found in the intestine of these diseased mice, and OA treatment protected against this loss. By preserving the mucus secreting goblet cells, OA ensured the normal mucus secretion and consequently the homeostasis between the host and the microenvironment.

We also detected that Gal-3, a galactoside-binding protein constitutively expressed in colonic epithelial cells, was extensively downregulated in the colon of EAE mice. A significant reduction of Gal-3 has also been reported in the inflamed mucosa from IBD patients, associated with epithelial breakdown and high intestinal levels of inflammatory markers [45]. It has been proposed that Gal-3, through its interaction with the cell surface-associated mucins, may contribute to the integrity of the mucosal barrier [46]. This would agree with our observation, since OA also preserves the expression level of Gal-3. Interestingly, in several experimental models, OA also protected the gastric mucosa against acute lesions [47].

Aware of the key role played by ROS in MS [48], we also analyzed the oxidative status of the GI tract in our model. We detected ROS overproduction in colon of EAE mice, as well as an elevated formation of lipid peroxidation products, while its antioxidant capacity, measured by a FRAP assay, decreased. However, these parameters with OA treatment remained close to control levels. Moreover, the expression of sestrin-3 was found to be downregulated in EAE. Sestrin-3 is a stress-responsive protein that reduces intracellular ROS accumulation and oxidative DNA damages. Transcriptional repression of sestrin family genes substantially increased ROS accumulation, thus pointing to these proteins as intrinsic antioxidant defenses [49]. Consistently, colon tissues from OA-treated EAE mice exhibited higher sestrin-3 levels than untreated-EAE mice in which ROS levels are found to be elevated. The association between oxidative stress markers and clinical sign severity score suggests role of colon oxidation in EAE disease. Cumulatively, our results show that the redox status was preserved in OA-treated EAE mice, and the protective effect of OA may be due, at least in part, to its antioxidant properties in the intestine. The correlation indexes between markers of oxidation, permeability, and clinical score suggest an intimate association between oxidative stress, leaky gut, and CNS disability, thereby opening new targeting pathways.

In addition, and in line with our previous findings in nervous tissues, EAE also resulted in upregulation of inflammatory cytokines TNFα, IL-1β, IL-23, and IL-17A and the chemokine KC, along with a significant reduction in the levels of IL-13, IL-33, and IL-17E, and the neurotrophin GDNF in colonic tissue. Neurotrophic factors, including GDNF, have been shown to play a crucial role in the pathogenesis of inflammation-induced intestinal barrier disruption [50].

IL-23 and IL-1β released from intestinal cells are essential for the differentiation of Th17 cells and the production of associated cytokines such as IL-17A, IL-22, and TNF-α. Concurrently, IL-17A production amplifies Th17 responses [51]. Besides, IL-17E is known to play a dual role by driving expression of Th2 cytokines such as IL-13, and simultaneously limiting the production of proinflammatory cytokines in the GI tract including IL-17A. Moreover, the loss of IL-17E results in enhanced pro-inflammatory cytokine production that is associated with an increased number of IL-17-, IFNγ-, and TNFα-producing T cells [52, 53]. Therefore, we may assume that the observed distorted network of cytokines and neurotrophic factors is associated with pro-inflammatory effector functions that alter intestinal epithelial integrity, thus weakening the barrier function in EAE-mice.

Interestingly, levels of IGF-1 decreased in serum of EAE mice, whereas its expression in colonic tissue tended to increase. Impaired IGF-1 concentration has been documented in gastrointestinal inflammatory disorders and treatment with IGF-1 improves intestinal barrier function [54, 55]. Thus, considering these evidences, our findings may support the hypothesis that EAE-induced impairment in IGF-1 levels contributes to the development of a leaky gut.

At the same time, high expression of IGF-1 in the colon of EAE mice together with elevated levels of inflammatory parameters was observed. Although the precise role of this local upregulation is not clear, studies from human and animal models of IBD propose hypotheses that include contribution to inflammation-induced fibrosis or mucosal healing [56]; further studies are needs to shed light on these proposals.

OA was previously found to abolish a harmful profile of cytokines at both systemic and local levels, as well as to block NF-κB activation [8, 10, 12, 57]. Therefore, another possible mechanism by which OA treatment might protect against EAE and its associated GI disturbances would be by preserving a healthy balance of pro-inflammatory-, anti-inflammatory-, and regulatory-cytokines/factors. In keeping with that, we found that OA treatment was effective in overturning the adverse bias linked to EAE disease.

Unexpectedly, the inflammasome NLRP6 was upregulated in colon tissue from EAE mice, whereas OA reduced its expression. Most studies to date have assigned a protective role to the activation of NLRP6 in various intestinal pathologies, with both intestinal inflammation and tumorigenesis significantly aggravated in the absence of NLRP6 [58]. However, a recent line of research has described that NLRP6 is upregulated in ileal Crohn’s disease, and a pathogenic role for NLRP6 in alloimmune-mediated intestinal damage has been proposed [59]. It seems that the regulation of NLRP6 expression/function depends on the biological context (e.g., under normal homeostatic vs during pathologic chronic inflammation) or even section of the GI tract studied [59, 60]. Future investigations are required to fully understand these differences.

Given the key role of intestinal epithelial cells (IECs) in the maintenance of intestinal homeostasis, our study has also verified the direct beneficial effects of OA in vitro in human IEC monolayers exposed to cytokines or injurious agents found to be overexpressed in EAE-mice colon. Currently, only a minority of studies have examined the modulating effects of triterpenes on the intestinal barrier functions, but several plant extracts containing triterpenes, such as boswellic acids, ursolic acid, or lupeolic acid improve epithelial barrier integrity and attenuate intestinal inflammation [61].

In line, we found that OA inhibited IL-8 expression and intracellular ROS production in Caco-2 cell monolayers exposed to different stressors. Moreover, OA protected the barrier integrity of the Caco-2 monolayers evidenced by its ability to prevent TEER drop, paracellular barrier opening and ZO-1 delocalization, under inflammatory conditions. ZO-1 proteins play a crucial role in maintaining the structure of the tight junctions and in regulating the epithelial barrier function. They appear as smooth arc-like structures, but under inflammatory conditions rearrange into an irregular appearance [62, 63]. We found that OA treatment preserved the structures of ZO-1, in concordance with its protective barrier function. OA was also protective against epithelial barrier opening in intestinal co-culture model in vitro, which has a similar cell composition to that of the human large intestine in vivo.

We have demonstrated that the presence of OA ameliorates the signs and symptoms of EAE induced mice, but the question remains as what the effect of OA is at the pre-EAE stage at the immune inflammatory level. With the limitations of a different model of autoimmune disease, experimental autoimmune myocarditis, we have observed that OA did not trigger significant differences in the proportion of CD3+, CD4+, and CD8+ T cells; CD14+ macrophages/monocytes; and CD19+ B cells; however, OA-treatment significantly increased the percentage/proportion of Treg, compared to control mice [12]. We expect a similar behavior in EAE, but this question remains for future investigations.

## Conclusion

In conclusion, and to the best of our knowledge, our study demonstrates for the first time that OA effectively regulates intestinal oxidative stress, inflammation, and intestinal integrity when administered to EAE mice. Since OA ameliorates MS classical clinical signs, this study remarks the probable relevance of the intestinal alterations in the evolution of the disease. Our data strongly support the therapeutic potential of OA for the treatment of MS and MS-related disorders.

## Supplementary Information

**Additional file 1: Figure S1.** Effect of OA treatment on clinical parameters in EAE mice. (A) Overview of the protocol of EAE induction and OA treatment. (B) Effect on the evolution of clinical signs and body weight (*n*= 15, in all groups). (C) Titers of anti-MOG_35-55_-IgG1 antibodies were evaluated by ELISA, in serum samples from mice of the different experimental groups. Results were expressed as the mean ± SEM, *n*=5-7 per group. *p<0.001 vs control; and ^‡^*p*<0.001 vs untreated-EAE. C, healthy mice. C+OA, healthy mice treated with OA. EAE, induced mice. EAE+OA, induced-mice treated with OA. **Figure S2.** The effect of OA on individual SCFA concentration. Proportions of individual short-chain fatty acids (SCFAs) in cecal samples from mice of the different experimental groups. (A) Relative distribution of individual straight-Chain Fatty Acids (%), and (B) Relative distribution of individual branched-Chain Fatty Acids, (%). Bar graphs represent the mean ± SEM of 5 animals. *p<0.001 vs control; and ^‡‡‡^*p*<0.05 vs untreated-EAE. C, healthy mice. C+OA, healthy mice treated with OA. EAE, induced mice. EAE+OA, induced-mice treated with OA. **Figure S3.** OA treatment modulates inflammatory parameters in serum from EAE mice. Levels of the inflammatory mediators TNFα, IL-1β, IL-23, IL-17, IGF-1, GM-SCF and galectin-3 in serum samples from mice of the indicated groups were quantified by commercial ELISAs. Results were expressed as the mean ± SEM, *n*=5-7 per group. *p<0.001, and ***p<0.05 *vs* control; and ^‡^*p*<0.001, ^‡‡^*p*<0.01 and ^‡‡‡^*p*<0.05 *vs* untreated-EAE. C, healthy mice. C+OA, healthy mice treated with OA. EAE, induced mice. EAE+OA, induced-mice treated with OA. **Figure S4.** Effect of iso-valeric acid treatment in intestinal epithelial cells. Caco-2 monolayers were treated for 24 h with the indicated doses of iso-valeric acid: (A) Cell viability, (B) intracellular ROS production and (C) IL-8 concentration in the cell-culture supernatant, are shown. (D) Differentiated Caco-2 cell monolayers were treated with iso-valeric acid at the apical side and transepithelial electrical resistance (TEER) was measured at 24h. TEER values normalized to the untreated control (100%) are shown. The assays were performed in duplicates, *n* = 3. Results were expressed as the mean ± SEM. ‡*p*<0.001, and ‡‡‡*p*<0.05 vs control.

## Data Availability

Data supporting the conclusions are presented in the manuscript. The datasets used and/or analyzed during the current study are available from the corresponding author on reasonable request.

## Abbreviations

AB: Alcian Blue
CNS: Central nervous system
DHE: Dihydroethidium
DMEM: Dulbecco’s modified Eagle’s medium
EAE: Experimental autoimmune encephalomyelitis
ELISA: Enzyme-linked immunosorbent assay
FRAP: Ferric reducing/antioxidant power
GDNF: Glial-derived neurotrophic factor
GI: Gastrointestinal
GM-SCF: Granulocyte-macrophage colony stimulating factor
iFABP: Intestinal fatty acid-binding protein
MDA: Malondialdehyde
MOG: Myelin oligodendrocyte glycoprotein
MPO: Myeloperoxidase
MS: Multiple sclerosis
OA: Oleanolic acid
PAS: Periodic acid-Schiff
ROS: Reactive oxygen species
SCFA: Short-chain fatty acids
O2^−^: Superoxide anion
t-BOOH: t-butyl hydroperoxide
TEER: Transepithelial resistance
TNFα: Tumor necrosis factor-α
ZO-1: Zonula occludens-1
