# Supplementary material for: Oleanolic acid ameliorates intestinal alterations associated with EAE
Source: J Neuroinflammation. 2020 Nov 27;17:363. doi: 10.1186/s12974-020-02042-6 (PMC7697371; doi:10.1186/s12974-020-02042-6)
Supplement: Supplementary file 1 — Additional file 1: Figure S1. Effect of OA treatment on clinical parameters in EAE mice. (A) Overview of the protocol of EAE induction and OA treatment. (B) Effect on the evolution of clinical signs and body weight (n= 15, in all groups). (C) Titers of anti-MOG35-55-IgG1 antibodies were evaluated by ELISA, in serum samples from mice of the different experimental groups. Results were expressed as the mean ± SEM, n=5-7 per group. *p<0.001 vs control; and ‡p<0.001 vs untreated-EAE. C, healthy mice. C+OA, healthy mice treated with OA. EAE, induced mice. EAE+OA, induced-mice treated with OA. Figure S2. The effect of OA on individual SCFA concentration. Proportions of individual short-chain fatty acids (SCFAs) in cecal samples from mice of the different experimental groups. (A) Relative distribution of individual straight-Chain Fatty Acids (%), and (B) Relative distribution of individual branched-Chain Fatty Acids, (%). Bar graphs represent the mean ± SEM of 5 animals. *p<0.001 vs control; and ‡‡‡p<0.05 vs untreated-EAE. C, healthy mice. C+OA, healthy mice treated with OA. EAE, induced mice. EAE+OA, induced-mice treated with OA. Figure S3. OA treatment modulates inflammatory parameters in serum from EAE mice. Levels of the inflammatory mediators TNFα, IL-1β, IL-23, IL-17, IGF-1, GM-SCF and galectin-3 in serum samples from mice of the indicated groups were quantified by commercial ELISAs. Results were expressed as the mean ± SEM, n=5-7 per group. *p<0.001, and ***p<0.05 vs control; and ‡p<0.001, ‡‡p<0.01 and ‡‡‡p<0.05 vs untreated-EAE. C, healthy mice. C+OA, healthy mice treated with OA. EAE, induced mice. EAE+OA, induced-mice treated with OA. Figure S4. Effect of iso-valeric acid treatment in intestinal epithelial cells. Caco-2 monolayers were treated for 24 h with the indicated doses of iso-valeric acid: (A) Cell viability, (B) intracellular ROS production and (C) IL-8 concentration in the cell-culture supernatant, are shown. (D) Differentiated Caco-2 cell monolayers wer [file 12974_2020_2042_MOESM1_ESM.pdf]

## Supplemental Legends

**Figure S1. Effect of OA treatment on clinical parameters in EAE mice.** (A) Overview of the protocol of EAE induction and OA treatment. (B) Effect on the evolution of clinical signs and body weight (*n*= 15, in all groups). (C) Titers of anti-$MOG_{35-55}$-IgG1 antibodies were evaluated by ELISA, in serum samples from mice of the different experimental groups. Results were expressed as the mean ± SEM, n=5-7 per group. *p<0.001 vs control; and ‡p<0.001 vs untreated-EAE. C, healthy mice. C+OA, healthy mice treated with OA. EAE, induced mice. EAE+OA, induced-mice treated with OA.

**Figure S2. The effect of OA on individual SCFA concentration.** Proportions of individual short-chain fatty acids (SCFAs) in cecal samples from mice of the different experimental groups. (A) Relative distribution of individual straight-Chain Fatty Acids (%), and (B) Relative distribution of individual branched-Chain Fatty Acids, (%). Bar graphs represent the mean ± SEM of 5 animals. *p<0.001 vs control; and ‡‡‡p<0.05 vs untreated-EAE. C, healthy mice. C+OA, healthy mice treated with OA. EAE, induced mice. EAE+OA, induced-mice treated with OA.

**Figure S3. OA treatment modulates inflammatory parameters in serum from EAE mice.** Levels of the inflammatory mediators TNFα, IL-1β, IL-23, IL-17, IGF-1, GM-SCF and galectin-3 in serum samples from mice of the indicated groups were quantified by commercial ELISAs. Results were expressed as the mean ± SEM, n=5-7 per group. *p<0.001, and ***p<0.05 *vs* control; and ‡p<0.001, ‡‡p<0.01 and ‡‡‡p<0.05 *vs*

untreated-EAE. C, healthy mice. C+OA, healthy mice treated with OA. EAE, induced mice. EAE+OA, induced-mice treated with OA.

**Figure S4. Effect of iso-valeric acid treatment in intestinal epithelial cells.** Caco-2 monolayers were treated for 24 h with the indicated doses of iso-valeric acid: (A) Cell viability, (B) intracellular ROS production and (C) IL-8 concentration in the cell-culture supernatant, are shown. (D) Differentiated Caco-2 cell monolayers were treated with iso-valeric acid at the apical side and transepithelial electrical resistance (TEER) was measured at 24h. TEER values normalized to the untreated control (100%) are shown. The assays were performed in duplicates, n = 3. Results were expressed as the mean ± SEM. ‡p<0.001, and ‡‡‡p<0.05 vs control.

**Con formato:** Resaltar

# Figure S1

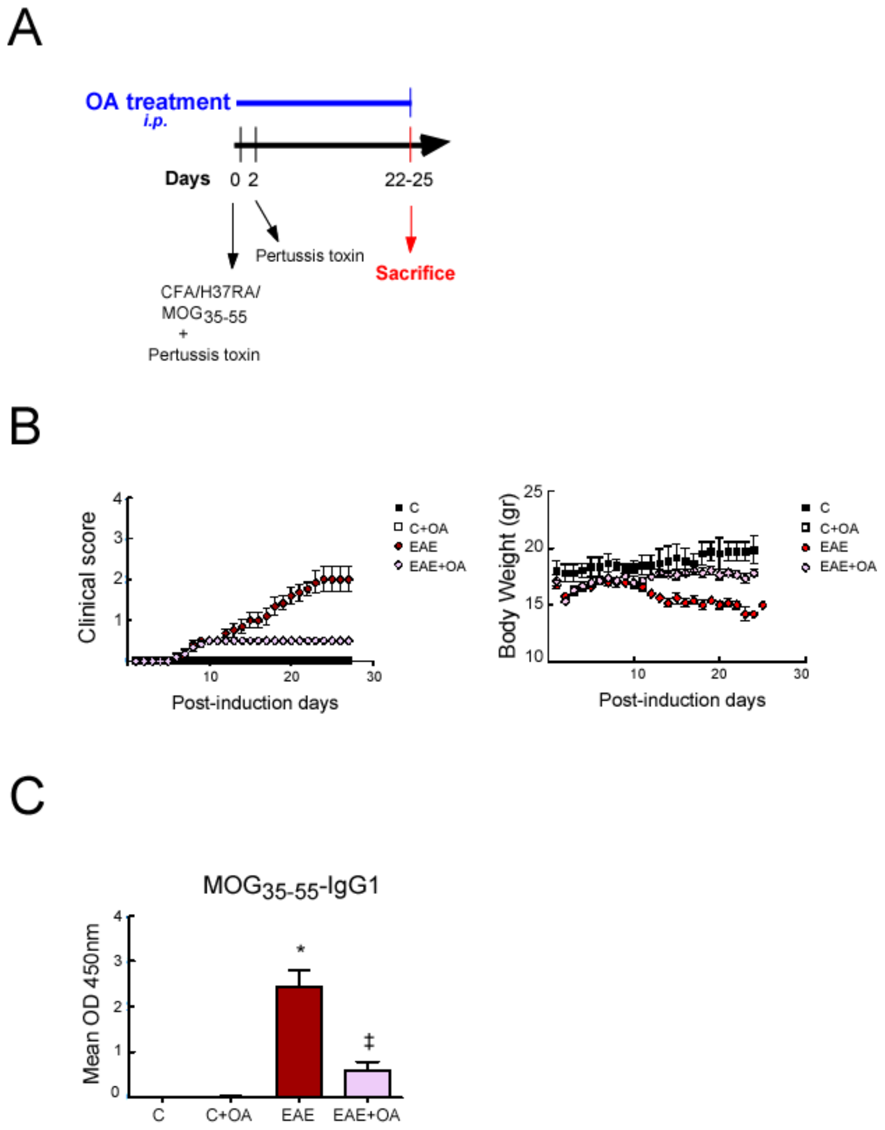


41

42

# Figure S2

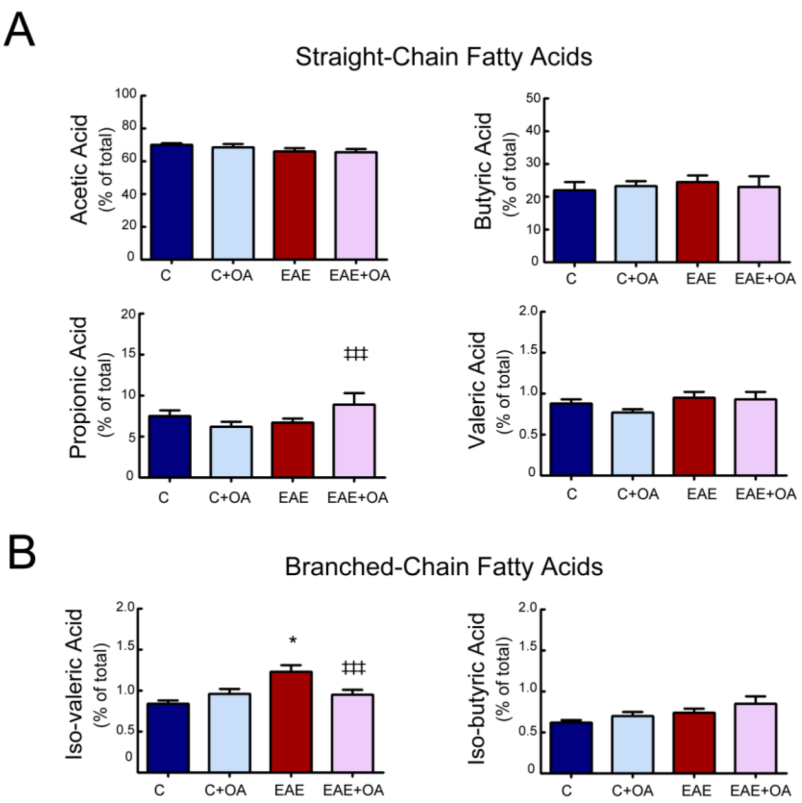
A
Straight-Chain Fatty Acids
Acetic Acid (% of total)
Butyric Acid (% of total)
Propionic Acid (% of total)
Valeric Acid (% of total)
‡‡
C
C+OA
EAE
EAE+OA
B
Branched-Chain Fatty Acids
Iso-valeric Acid (% of total)
Iso-butyric Acid (% of total)
*
‡‡

# Figure S3

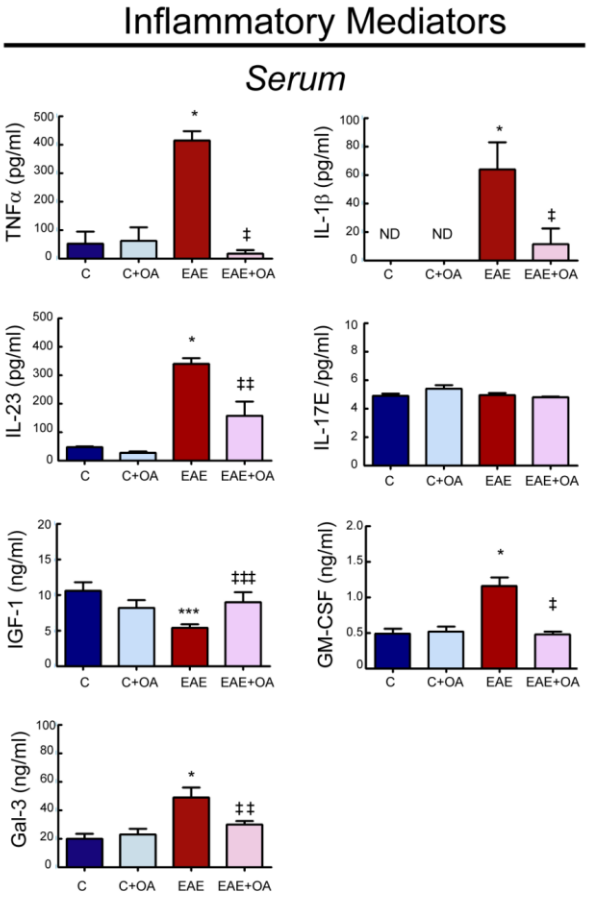
Inflammatory Mediators
Serum
TNFα (pg/ml)
0
100
200
300
400
500
*
‡
IL-1β (pg/ml)
0
20
40
60
80
100
ND
ND
*
‡
IL-23 (pg/ml)
0
100
200
300
400
500
*
‡‡
IL-17E /pg/ml)
0
2
4
6
8
10
IGF-1 (ng/ml)
0
5
10
15
20
***
‡‡‡
GM-CSF (ng/ml)
0
0.5
1.0
1.5
2.0
*
‡
Gal-3 (ng/ml)
0
20
40
60
80
100
*
‡‡
C
C+OA
EAE
EAE+OA

# Figure S4

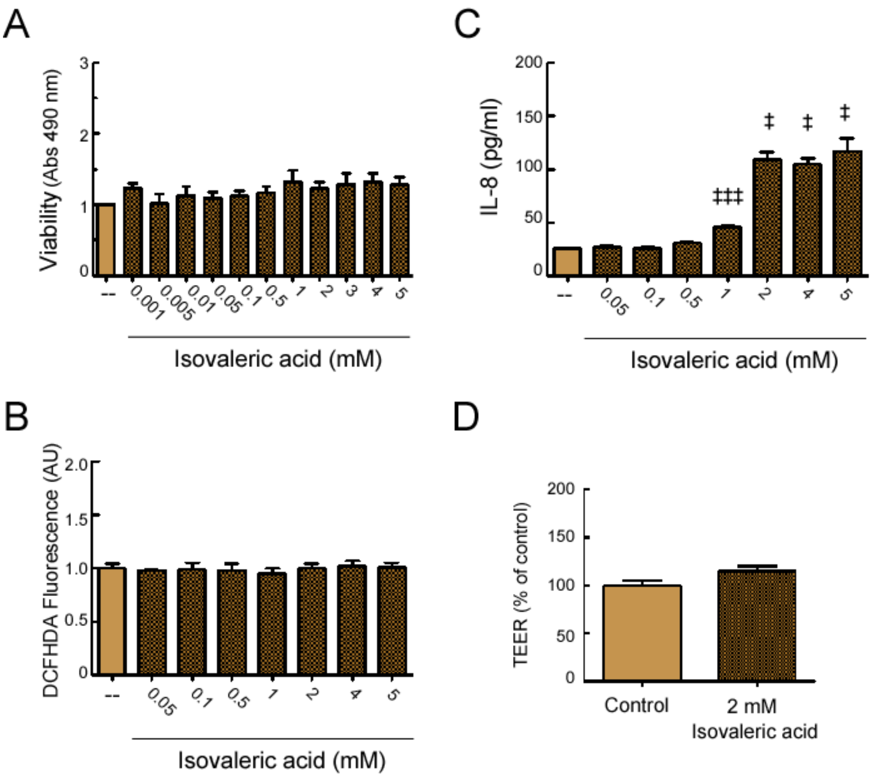
A
Viability (Abs 490 nm)
3
2
1
0
--
0.001
0.005
0.01
0.05
0.1
0.5
1
2
3
4
5
Isovaleric acid (mM)
C
IL-8 (pg/ml)
200
150
100
50
0
‡‡‡
‡
‡
‡
--
0.05
0.1
0.5
1
2
4
5
Isovaleric acid (mM)
B
DCFHDA Fluorescence (AU)
2.0
1.5
1.0
0.5
0
--
0.05
0.1
0.5
1
2
4
5
Isovaleric acid (mM)
D
TEER (% of control)
200
150
100
50
0
Control
2 mM
Isovaleric acid
